# The Impact of Wearable Device Enabled Health Initiative on Physical Activity and Sleep

**DOI:** 10.7759/cureus.825

**Published:** 2016-10-11

**Authors:** Olga Crowley, Laura Pugliese, Stan Kachnowski

**Affiliations:** 1 Innovation Research, Healthcare Innovation and Technology Lab, Inc.; 2 Department of Management Studies, Indian Institute of Technology - New Delhi; 3 New York Psychiatric Institute, Columbia University Medical Center; 4 Ryder Trauma Center, University of Miami, Medical School; 5 HITLAB, Healthcare Innovation & Technology Lab

**Keywords:** wearable technology, digital health, employee wellness, physical activity, sleep

## Abstract

**Objectives:**

The Personal Health Management Study (PHMS) is an assessment of the effect of a voluntary employee-facing health initiative using a commercially-available wearable device implemented among 565 employees of Boehringer Ingelheim Pharmaceuticals, Inc. The results of the initiative on physical activity (measured as steps) and sleep is reported.

**Methods:**

This was a 12-month, prospective, single-cohort intervention study using a wearable activity-measuring device tracking steps and sleep (entire study period) and a system of health-promoting incentives (first nine months of study period). The findings from the first nine study months are reported.

**Results:**

The mixed model repeated measures approach was used to analyze the data. There was no significant difference in steps between the first month (7915.6 mean steps per person per day) and the last month (7853.4 mean steps per person per day) of the intervention. However, there was a seasonal decline in steps during the intervention period from fall to winter, followed by an increase in steps from winter to spring. In contrast, sleep tended to increase steadily throughout the study period, and the number of hours slept during the last month (7.52 mean hours per person per day) of the intervention was significantly greater than the number of hours slept during the first month (7.16 mean hours per person per day).

**Conclusions:**

The impact of the initiative on physical activity and sleep differed over the period of time studied. While physical activity did not change between the first and last month of the intervention, the number of hours slept per night increased significantly. Although seasonal changes and study-device habituation may explain the pattern of change in physical activity, further evaluation is required to clarify the reasons underlying the difference in the impact of the initiative on the dynamics of steps and sleep.

## Introduction

Physical inactivity and sleep deprivation have long been established as predictors of all-cause morbidity and mortality. Recently, the contribution of physical inactivity to all-cause mortality was reported to be comparable to that of smoking [1]. While smoking causes approximately five million deaths worldwide, inactivity has been linked to 5.3 million, and decreasing inactivity by 10% or 25% can prevent more than 1.3 million deaths annually [1]. Furthermore, physical inactivity explains approximately six percent of cardiovascular morbidity, seven percent of disease burden from type 2 diabetes, 10% of breast cancer, 10% of colon cancer, and nine percent of premature mortality worldwide [1]. According to the Centers for Disease Control and Prevention (CDC), approximately 80% of adults in the United States (US) do not meet recommended guidelines for physical activity [2]. Moreover, the average number of steps per day for adults living in the US is 5,117, which is significantly lower than for their peers living in Switzerland, western Australia, and Japan who average 9,695, 7,168, and 9,650 daily steps, respectively [3]. Like physical inactivity, sleep deprivation is common in the US. Large epidemiological studies have reported that approximately one-third of US adults suffer from sleep deprivation, which interferes with their daily functioning and increases their risks for cardiovascular disease, obesity, and diabetes [4-5]. Systematic reviews of prospective studies identified sleep deprivation as a predictor of all-cause [6] and coronary heart disease mortality and morbidity [7]. Therefore, promoting physical activity and healthy sleep habits is an important public health imperative.

The burgeoning field of healthcare information technology has produced a wide range of mobile devices that allow for comprehensive assessment of one’s activity, including measurement of steps taken and hours of sleep attained. In addition to passively tracking activity data, these devices allow one to set daily goals and send reminders and feedback messages. Using activity monitoring devices—even the simplest ones, such as pedometers—has been shown to increase physical activity [8]. In fact, a recent meta-analysis showed that the use of mobile devices is associated with significant increases in physical activity levels [9]. Employee wellness programs that use wearable activity monitoring devices to promote physical activity and healthy sleep are becoming increasingly popular, as these interventions may reduce disease burden, absenteeism, and healthcare costs, while enhancing productivity and morale [10-11]. It has been projected that in the next five years, more than 13 million wearable mobile devices will be integrated into corporate wellness plans [12]. However, evidence on the impact of these devices in the context of corporate wellness programs has been mixed. The RAND Corporation has recently reported no statistically significant clinical or financial benefits [13]. There is some evidence that factors such as program duration of 12 months or longer and individual tailoring with personalized activity goals may help increase program effectiveness [14].

In September 2013, Boehringer Ingelheim Pharmaceuticals, Inc. (BI) conducted the Personal Health Management Study (PHMS), a 12-month employee-facing voluntary health initiative and research study. This program was implemented by the Healthcare Innovation and Technology Lab, Inc. (HITLAB). The primary goal of PHMS was to encourage employees to increase their physical activity levels (measured as daily step average) and hours of sleep per night. The program “intervention” consisted of providing employees with the UPTM (Jawbone, San Francisco, CA), a wrist band that tracks steps and sleep data; and using a variety of incentives to encourage them to use and adhere to use of the UPTM and engage in positive health behaviors. We hypothesized that this program would produce significant increases in physical activity and sleep among all study participants. Specifically, we expected to observe a significant increase in steps and number of hours slept per night when measuring the difference in these outcomes between the first month and the last month of the intervention, as well as the differences between consecutive months throughout the entire study period.

## Materials and methods

### Participants

565 BI employees aged 23–67 drawn from a population of approximately 3,500 eligible individuals at BI’s Ridgefield, Connecticut campus were recruited and consented into the PHMS. The inclusion criteria for this program included permanent employment at BI and access to a device capable of hosting the Jawbone UP® mobile application (including the iPhone, Android phone, iPad, or iPod touch). Individuals who were pregnant, had an ambulatory disability, terminated employment at BI, or previously owned an UP® by Jawbone band were not eligible for this study. The study was approved by the Chesapeake Institutional Review Board. 

### Measures

Physical activity and sleep - UP® by Jawbone is a consumer-facing activity measuring device that uses a precision motion sensor and actigraphy monitoring to track steps and sleep. The study participants synced their UP® band to the UP® iPhone application (UP® app) that displayed their daily steps and sleep (captured as hours of deep and light sleep, and total number of hours slept) data, provided weekly and monthly reports, and sent messages encouraging the participants to meet their respective goals. 

The PHMS survey was a quarterly questionnaire that included 47 questions drawn from several established instruments, including the SF-36 [15], Brunel Lifestyle Physical Activity Questionnaire [16], Kaiser Physical Activity Survey [17], Sleep Heart Health Study Sleep Habits Questionnaire [18], Functional Outcomes of Sleep Questionnaire [19], RAND 36-Item Health Survey 1.0 Questionnaire [20], Pittsburgh Sleep Quality Index [21], and the 21-item disease checklist from the Midlife in the United States (MIDUS) study [22]. The survey also captured demographic information, health behaviors (smoking, exercise/physical activity levels, sleep), diseases, life events, social support, and perceived health. This survey has not been previously validated. For the purposes of the present report, only demographic information from this survey (age and sex) was included in the analysis. Height and weight was measured for all participants using objective assessments.

Study Design and Procedures

The PMHS utilized a 12-month prospective, single-cohort study design. The intervention included wearing the UP® band, which tracked physical activity (steps) and sleep (hours). In addition to tracking physical activity and sleep, the band provided participants with the ability to set up daily step and sleep goals. The UP® application, which was installed on the participants’ smart phone, sent messages to the participants that encouraged them to meet their individual goals and reported their progress. All participants received a training on how to use the device and app at the time of enrollment, and devices were calibrated to participants at this time. In addition to wearing the UP® band, the intervention included sending participants weekly emails providing aggregated data about physical activity and sleep of the study population; sending participants weekly e-mails providing health tips; providing participants with a series of virtual challenges and awards that included digital badges, prizes, and charitable donations; and providing opportunities to participate in voluntary on-campus social events.

During the course of the study, the participants were invited to attend quarterly study visits at Baseline and Months three, six, nine, and 12. In the first appointment, the participants were enrolled in the study, instructed on the use of the study device, and were requested to fill out a survey that captured demographic information and information on current health behaviors and psychosocial functioning. The participants also had their BMI measured at this appointment by certified nursing assistants. Step and sleep data were subsequently collected by the UP® band for a 12-month period. During the study period, the participants were invited to the second through fifth appointments on a quarterly basis via email. The e-mails invited them to schedule a check-in appointment during the first two weeks of each quarter in which data was being collected from the UP® band. The second and third appointments were conducted in person and included completion of PHMS survey and measurement of BMI. The fourth and fifth appointments were conducted virtually and only included completing the PHMS survey.

Data Analysis

As the study commenced on September 25, 2013, thereby leaving only six days of the first study month, we did not include data from September in the analysis. Additionally, the system of health-promoting incentives terminated after the first nine months of the study period (June 2014), thereby limiting the intervention to simply wearing the UP® band. In order to eliminate the potential for confounding of the results by changing the intervention after nine months, the present manuscript reports the findings only from the first nine months of the program (October 2013–June 2014).

Physical activity and sleep data were analyzed separately. Steps were measured as daily number, and sleep was evaluated as total number of hours per night. The number of hours of deep and light sleep was not included in the analysis as these measures may need to be further investigated to be considered valid and reliable estimates of sleep quality. For both steps (measured as daily number) and sleep (measured as number of hours per night) the data for that measure were averaged for each study month. The mixed model repeated measures (MMRM) approach was used to analyze data. We fitted the model that included study month, subject ID number, baseline steps/sleep levels, study month x baseline interaction, age, sex, BMI, age x sex interaction, and age x BMI interaction as predictors of steps or sleep. Age, baseline steps/sleep levels, and BMI were entered in the model as continuous variables, while sex and study month were entered as categorical variables.

Monthly changes in steps and sleep comparing consecutive study months and the first month of the study to the last one were examined using pairwise comparison (supported by the SAS procedure MIXED and its LSMEANS statement). As previous studies showed considerable impact of seasonal changes on physical activity [23-24], we compared aggregated data for fall 2013, winter 2013-2014, and spring 2014. Fall was defined as October and November 2013; winter was defined as aggregated data from December 2013 and January and February 2014; spring was defined as March, April, and May 2014; and summer was defined as June 2014. We used LSMESTIMATE statement to conduct this comparison [25-26]. To correct for Type I error, we used Bonferroni adjustments. We adjusted each analysis by the number of comparisons among the months or seasons. For comparing changes across consecutive study months and the first month of the study to the last one, we used 0.05/9 = 0.006; for comparing data aggregated across seasons, we used 0.05/5 = 0.01. Three hundred twenty three participants had missing data for at least one month during the study period. To determine the impact of missing data on our findings, we conducted sensitivity analyses using the last observation carried forward (LOCF) approach and found that imputing missing data did not alter our results. The findings from the missing data analysis are briefly described in the Results section.

## Results

**Figure 1:**
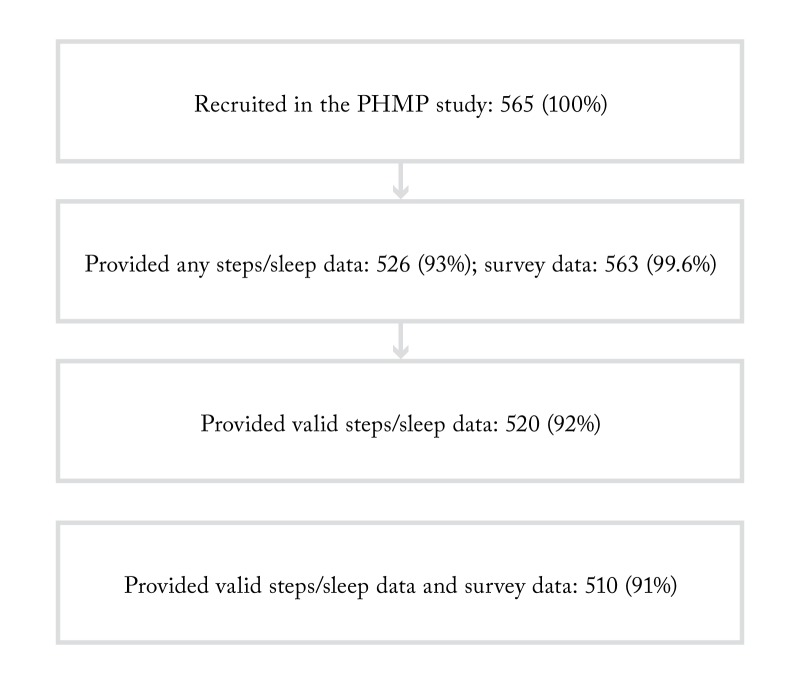
Defining final sample size included in the data analysis

From the total of 565 enrolled participants, 526 (93%) provided steps/sleep data, of whom 520 (92%) participants provided data that was greater than zero for steps and no less than two and no greater than 13 hours for sleep, a range established based on a literature review of reported sleep duration values in order to eliminate spurious data for sleep time [27-30]. A total of 563 (99.6%) participants provided survey data. A total of 510 (91%) participants had both survey and valid steps/sleep data. Therefore, our final sample size consisted of 510 participants. Table 1 describes clinical and demographic characteristics of the study sample. Figure 1 summarizes the process of finalizing the study sample.

**Table 1:** The sample’s demographic and clinical characteristics (N=510)

Variable		N (%)	Range	Mean (SD)
Age		506 (99%)	23-67	43.46 (9.20)
Gender	Male	232 (45%)		
	Female	278 (55%)		
BMI (Body Mass Index)		503 (99%)	18.11-51.37	27.66 (5.34)
Chronic diseases	Blood clots	8 (2%)		
	Heart disease	7 (1%)		
	Heart murmur	28 (5%)		
	Diabetes	17 (3%)		
	Circulation problems	5 (1%)		
	TIA or stroke	3 (.6%)		
	Anemia or other blood disease	26 (5%)		
	Depression	0 (0%)		
	Cholesterol problems	84 (16%)		
	Asthma	43 (8%)		
	Thyroid disease	23 (5%)		
	Peptic ulcer disease	5 (1%)		
	Cancer	12 (2%)		
	Colon polyp	28 (5%)		
	Insomnia	8 (2%)		
	Restless legs syndrome	1 (.2%)		
	Sleep apnea	13 (3%)		
		510 (100%)		

### Physical activity

The number of steps did not change significantly when comparing the first month and last month of the study period (t=-0.6, p=0.55). However, there were significant changes from month-to-month at some points during the study period (F (8, 3106) = 7.48, p<.0001; please see Table 2 for the significance of all fixed effects tested in the model). As shown in Table 2, these changes were affected by the baseline steps levels. Finally, there were significant age and sex differences in the steps during the study period. No other demographic differences reached statistical significance (see Table 2).

**Table 2:** Significance of fixed effects tested in the model (Type 3): Steps

Effect	Num DF	Den DF	F Value	Pr > F
Study month	8	3106	7.48	<.0001
Baseline steps	1	509	1605.83	<.0001
Baseline steps x study month	8	3096	15.03	<.0001
Age	1	490	2.89	0.0898
Sex	1	509	3.73	0.0541
BMI	1	487	0.24	0.6253
Age x sex	1	504	5.82	0.0162
Age x BMI	1	487	0.98	0.3236

Monthly Step Changes

Table 3 shows mean monthly step values that are adjusted for all fixed effects in the model and significance tests for the consecutive monthly means. Significant monthly changes are highlighted in bold. There was a consistently significant decline in steps from October 2013 to January 2014, followed by significant increases from January 2014 to February 2014, March 2014 to April 2014, and April 2014 to May 2014. When we compared the first month of the intervention (October 2013) with the last (June 2014), there was no significant difference (t=-0.6, p=0.55).

**Table 3:** Average steps per month

Study Month	Mean	Standard Error	Δ*	p
Oct-13	7915.6	79.763	-	-
Nov-13	7601.6	81.01	-314	0.0004
Dec-13	7140.3	83.5	-461.4	<.0001
Jan-14	6787.5	84.9	-352.74	0.0002
Feb-14	7101.1	87.14	313.58	0.0012
Mar-14	7179.7	89.88	78.59	0.4338
Apr-14	7526.9	91.22	347.24	0.0008
May-14	7897.8	93.47	370.9	0.0005
Jun-14	7853.4	98.01	-44.4	0.6912
*Δ describes the difference between two consecutive months (significance at .006 level)				

Seasonal Step Changes

The decline from fall to winter (D = -749, t=-12.57, p<.0001), and the increases from winter to spring (D = 525, t=8.96, p<.0001) and spring to summer (June 2014) (D =318.59, t=3.45, p<.0001) were significant. Comparisons of fall 2013 to spring 2014 revealed significantly greater number of steps in the fall (D = 223.81, t=3.54, p<.0001), and comparison of fall 2013 to June 2014 showed comparable number of steps (D = -94.78, t= -1.0, p=0.3158). As shown in Table 2, age x sex interaction and baseline step levels were significantly associated with steps during the study period (F(1,504)=5.82, p=0.0162). To interpret the significant interaction between age and sex, we created three age groups that represented younger (23–38 years, n=165), middle-aged (39–47 years, n=170), and older (48–67 years, n=171) participants. These cut-off age values were selected because they allowed for categorizing study participants into three groups that were similar in size and significantly different with respect to their respective average age values. This categorization was supported by the SAS procedure RANK. Table 4 describes average steps for the three age groups and both genders, and the differences in steps between men and women within each age group.

**Table 4:** Average steps among young, middle-aged, and older men and women

Age Groups	Sex	Mean	Std Dev	Δ
23-38 years	Male (n=86)	6671.59	2384.94	864.01
	Female (n=79)	7535.6	2607.55	
39-47 years	Male (n= 84)	7693.07	3385.71	138.78
	Female (n= 86)	7831.85	2836.55	
48-67 years	Male (n= 59)	7290.58	3663.77	219.3
	Female (n= 112)	7509.88	3144.99	

As Table 4 demonstrates, while female sex was associated with greater physical activity, the difference between men and women was the largest among the youngest participants. Age group 39–47 had the greatest number of steps.

### Sleep

The number of hours slept changed significantly during the study period (F (8, 2943) = 28.31, p<.0001; please see Table 5 for the significance of all fixed effects tested in the model). As shown in Table 5, these changes were affected by the baseline sleep levels. Finally, there were no demographic differences in the patterns of sleep change during the study period. 

**Table 5:** Significance of fixed effects tested in the model (Type 3): Sleep

Effect	Num DF	Den DF	F Value	Pr > F
Study month	8	2943	28.31	<.0001
Baseline steps	1	503	484.59	<.0001
Baseline steps x study month	8	2942	23.94	<.0001
Age	1	443	0.15	0.6958
Sex	1	468	2.32	0.1285
BMI	1	440	0.02	0.8958
Age x sex	1	462	1.4	0.2369
Age x BMI	1	439	0.01	0.9123

Monthly Sleep Changes 

Table 6 shows average monthly sleep adjusted for all fixed effects in the model and significance tests for the consecutive means.

**Table 6:** Average hours of sleep per month *Δ describes the difference between two consecutive months (significance at .006 level)

Study Month	Mean	Standard Error	Δ*	p
Oct-13	7.16	0.04	-	-
Nov-13	7.19	0.04	0.03	0.5368
Dec-13	7.35	0.04	0.16	0.0006
Jan-14	7.34	0.04	-0.01	0.8689
Feb-14	7.42	0.04	0.08	0.1234
Mar-14	7.50	0.04	0.08	0.1515
Apr-14	7.58	0.04	0.08	0.1377
May-14	7.59	0.05	0.01	0.8607
Jun-14	7.52	0.05	-0.07	0.2521

As Table 6 demonstrates, there was a steady increase in hours of sleep throughout the study period, with the exception of change from December to January and May to June. The increase in number of hours of sleep from November to December was significant. During the last month of the intervention (June 2014), participants slept significantly greater hours per night than during the first month of the intervention (October 2013) (D= 0.18, t=3.85, p=0.0001). 

Seasonal Sleep Changes

The increase from fall to winter (D = 0.19, t=6.38, p<.0001) and winter to spring (D = 0.18, t=5.93, p<.0001) was significant.

Missing Data Analysis

We imputed missing data using LOCF approach and then repeated our analysis. Testing the model that predicted average steps per month revealed significant effects of study month (F(8, 492)=23.15, Chi-Square = 187.85, p<.0001, p<.0001), baseline steps (F(4,497)= 227.86, Chi-Square = 97.59, p<.0001, p<.0001 ), and study month x baseline steps interaction (F (32,1266)=3.0, Chi-Square=95.86, p<.0001, p<.0001). We observed a seasonal decline in steps in the fall and winter followed by an increase from winter to spring. Testing the model that predicted average sleep per month revealed significant effects of study month (F(8, 387)=5.06, Chi-Square = 41.24, p<.0001, p<.0001), baseline sleep (F(3,389)=73.37, Chi-Square = 220.12, p<.0001, p<.0001 ), and study month x baseline sleep interaction (F (24,826)=4.13, Chi-Square=101.27, p<.0001, p<.0001). We observed a steady increase in sleep throughout the entire study period, with an exception of a slight decline from May to June. In summary, using all available data and LOCF produced similar results.

## Discussion

Physical activity showed no difference in steps between the first and last months of the program. However, there were significant seasonal changes during the study period. The seasonal changes included a decline in steps from fall 2013 to winter 2013/2014, followed by an increase in steps in spring 2014. The seasonal variations in physical activity observed in our study are consistent with previous studies that have found a decline in steps during cold and wet months [31-33]. However, given that the first (October) and last (June) months of our study both were in seasons with weather conducive to physical activity in our region, seasonal variation in physical activity would not seem to explain the absence of a significant change in steps from the first to the last month of the study. One potential contributor to the observed step dynamics may be habituation to the study device. Specifically, by the end of the study period when the weather became conducive to physical activity, the participants may have become used to wearing the band so it could not trigger any changes in steps. This possibility is consistent with the transtheoretical model of health behavior change [34] that identifies six stages of behavior modification: pre-contemplation, contemplation, preparation, action, maintenance, and termination. To maintain the initial favorable behavioral change, additional efforts are required; otherwise it will terminate. Thus, the absence of a significant difference between the first and the last months of the intervention may be explained by the failure of the program to maintain motivation for change.

The dynamics of sleep during the study period revealed a different pattern than seen with steps. Sleep steadily increased throughout the study period and average sleep duration was significantly greater in the end of the intervention than in the beginning. This effect was observed among all participants regardless of their age and gender.

With few exceptions, the average number of hours slept per night increased throughout the entire study period, and there was a significant increase in sleep from the first to the last month of the intervention. Therefore, neither seasonal changes nor transtheoretical model may explain the impact of the intervention on sleep. One contributor to the discrepancy of steps and sleep dynamics may be the difference in the underlying mechanisms that drive these health behaviors. For example, establishing a regular sleep/wake schedule is the key to improving sleep hygiene, and as a consequence, increasing sleep duration [35-36]. The intervention may have had greater impact on factors that help establish sleep/wake schedule and improve sleep hygiene such as self-monitoring [35], knowledge of sleep hygiene [36], and increased overall awareness of the importance of sleep. It also may have been possible that prior to participation in the PHMP study, the participants were less concerned about their sleep than about their physical activity. Therefore, there might have been greater room for improvement in sleep duration due to increased awareness.

Our findings are inconsistent with the previous literature that overall reports mixed evidence of the association of physical activity and sleep, but frequently indicates that there is a positive association between physical activity and sleep [37-42]. Sleep duration is a strong predictor of physical activity [43]. In fact, regular aerobic exercise training has been suggested as a treatment for improving sleep [44]. Thus, positive changes in sleep are not consistent with the lack thereof in steps. We did not, however, investigate changes in steps and sleep for the same individual; rather, we analyzed steps and sleep separately. Investigating the consistency of steps and sleep dynamics within the same individual may help clarify this issue.

Our study contributes to the previous literature in three ways. First, few workplace wellness programs targeted both physical activity and sleep; their focus has been on physical activity, diet, mental health, and physiological markers, and they did not include sleep [45]. The few interventions that involved sleep education did not target sleep as a primary outcome [46]. Importantly, we measured both physical activity and sleep using objective assessments (instead of relying on self-report data). This assessment was possible because PHMS used a device that measures both steps and sleep, while previous interventions tended to use pedometers [47]. While helpful in increasing physical activity, pedometers neither measure sleep nor provide active feedback regarding daily physical activity and sleep goals. Of note, actigraphy has been recommended by the American Academy of Sleep Medicine to measure sleep outcomes [48]. Third, the extended duration of our intervention and coverage of multiple seasons (fall, winter, spring, and the first month of summer) allowed us to evaluate the effect of seasonal changes on physical activity and sleep.

Our study has six methodological limitations. First, the absence of a control group limits the interpretation of our findings. Second, baseline data without intervention for physical activity and sleep was not available. Obtaining this data would have required wearing the study device, which in itself was part of the intervention. Third, our sample consisted of BI employees who have high socio-economic status (SES). SES is positively associated with health-promoting behaviors [49], which may have confounded our findings. Fourth, self-selection bias may have influenced our results as the employees who were interested in improving their physical activity and sleep levels would have been more likely to participate in the program. Fifth, it may be difficult to disentangle the effects of the season and the intervention on physical activity. Finally, it was not possible to assess weather-related changes in indoor physical activities, such as going to a gym or participation in winter activities, such as cross-country skiing, skating, and ice hockey [50]. These activities may have been substitutes for walking during the cold and wet months. In contrast to physical activity that may take many forms, sleep was evaluated in terms of duration. Thus, the completeness of measurement may have differed for the two study outcomes. Future studies should assess physical activity comprehensively and use control groups. It would also be informative to evaluate the amount of sedentary time, which has recently been reported to predict all-cause mortality and morbidity regardless of physical activity levels [51].

## Conclusions

In summary, we found that using a novel healthcare technology device in a large voluntary employee health initiative led to a significant increase in hours slept, but no significant change in physical activity, among employees. The discrepancy between steps and sleep dynamics observed in our study highlights the importance of targeting multiple health behaviors as outcomes of wellness interventions. Indeed, studies show that physical activity and sleep may independently predict health outcomes, such as maintaining healthy weight [51-55]. Therefore, our findings may inform future workplace interventions aimed at promoting employee wellness. Further analyses that may clarify our findings include identifying the demographic groups that benefitted the most and the least from the intervention and investigating the association between steps and sleep dynamics.
